# Overview of Interim and Temporary Restorations of Teeth During Endodontic Treatment

**DOI:** 10.7759/cureus.60591

**Published:** 2024-05-19

**Authors:** Pratik Rathod, Aditya Patel, Anuja Ikhar, Manoj Chandak, Shwetana Kurundkar, Lalit Pawar, Shefali Singh, Paresh Pawar, Khyati Manik

**Affiliations:** 1 Department of Conservative Dentistry and Endodontics, Sharad Pawar Dental College and Hospital, Datta Meghe Institute of Higher Education and Research, Wardha, IND; 2 Department of Orthodontics, Datta Meghe Institute of Higher Education and Research, Wardha, IND; 3 Department of Pediatric and Preventive Dentistry, Pacific Dental College and Hospital, Pacific Academy of Higher Education & Research University, Udaipur, IND

**Keywords:** provisionalization, interim restoration, temporary restoration, root canal treatment, endodontics

## Abstract

Root canal treatment of vital, non-infected teeth can often be completed in a single visit, negating the necessity for dressing and provisionalization. Conversely, cases involving infected canals typically demand multiple visits, during which antibacterial medicaments are applied, making effective provisionalization crucial for varying durations. The key components of a successful root canal treatment include adequate canal shape to promote efficient obturation, thorough chemical and mechanical debridement, and complete removal of pulp tissue remnants and bacteria. The primary cause of pain following the initiation of endodontic treatments is often attributed to inadequate debridement or incomplete removal of the pulp tissue, closely followed by insufficient temporary restorations. This review aims to comprehensively overview provisionalization materials used during and immediately after endodontic procedures.

## Introduction and background

**Figure 1:**
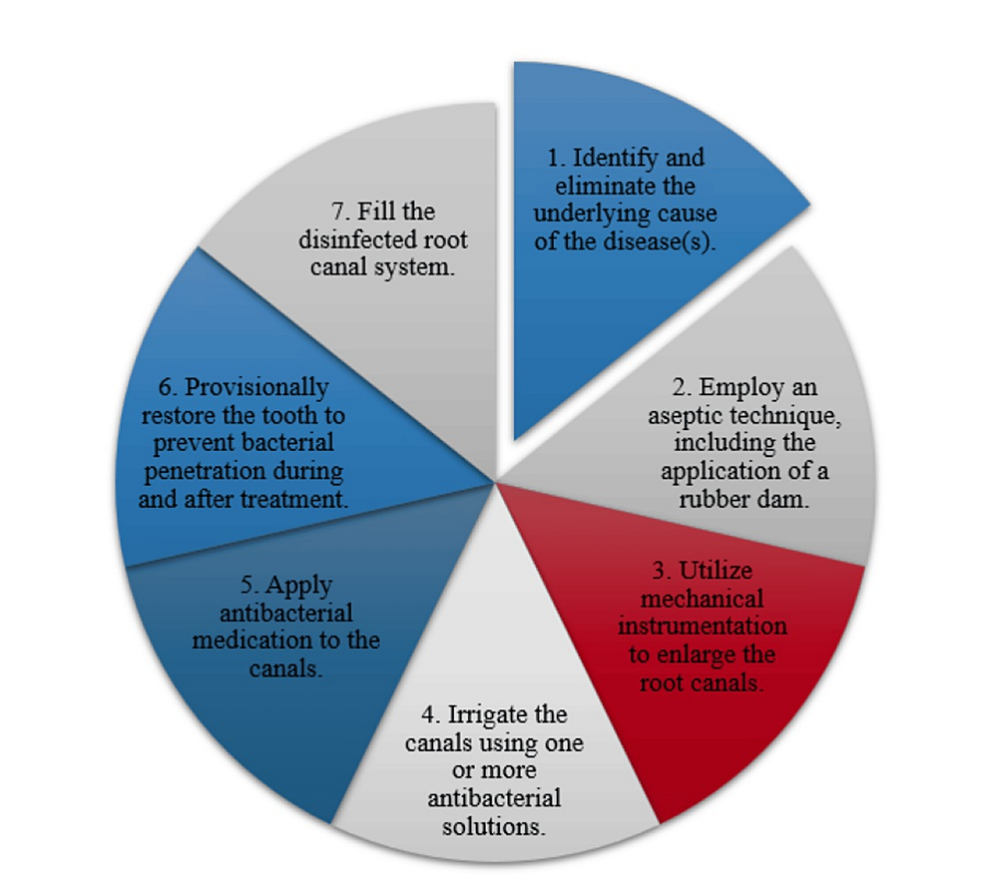
Comprehensive endodontic treatment regimen: eradicating microbial flora in tooth root canal systems Image credit: Pratik Rathod.

Most pulp and periapical disorders are caused by bacteria found in the tooth, especially in the root canals [1]. Therefore, the primary objectives of endodontic therapy should focus on eradicating bacteria from the canal [1]. Following that, upholding the tooth's disinfection state is essential for preventing bacterial ingress both during and after treatment. Reaching this objective would allow the tooth to remain in the oral cavity and return to its normal functional and anatomical status. The eradication of microbial flora within a tooth and its root canal system typically entails eight steps, constituting a standard endodontic treatment regimen commonly employed by dentists (Figure 1) [1].

Failure to include the steps above in the treatment protocol may lead to the survival and multiplication of existing bacteria within the tooth or the introduction of new organisms, potentially resulting in the establishment of bacterial colonies. Ultimately, these scenarios could perpetuate the existing apical periodontitis or give rise to new lesions. Therefore, to facilitate favourable healing of the apical periodontium after endodontic therapy, the treatment plan must incorporate all of the previously stated procedures, with each step following recognized and verified protocols. The primary cause of pulp and periapical disorders is the existence of microbes inside the tooth [2]. Therefore, physicians must identify how the microbes entered the tooth to remove the entry pathway and stop more bacterial infiltration. Bacterial infiltration into teeth can occur primarily through cavities, exposed dentine, cracks, and poor restorative margins [2-4].

Recent laboratory research indicates that within a few days of exposure to saliva, substantial leakage occurs at the coronal portion of the root canal filling, ranging from 33% to 85% of the total length of the root [5,6]. Insufficient temporary restorations during endodontic treatment rank as the second most significant factor contributing to ongoing pain after treatment initiation, following inadequate debridement or incomplete removal of the pulp tissue, which stands as the primary cause [7]. To ensure successful interim provisionalization between appointments, it is crucial to possess a comprehensive understanding of materials. This knowledge enables meeting diverse clinical needs, including time constraints, occlusal stresses, wear resistance, access complexity, and preservation of tooth structure.

## Review

Temporary restoration

**Figure 2:**
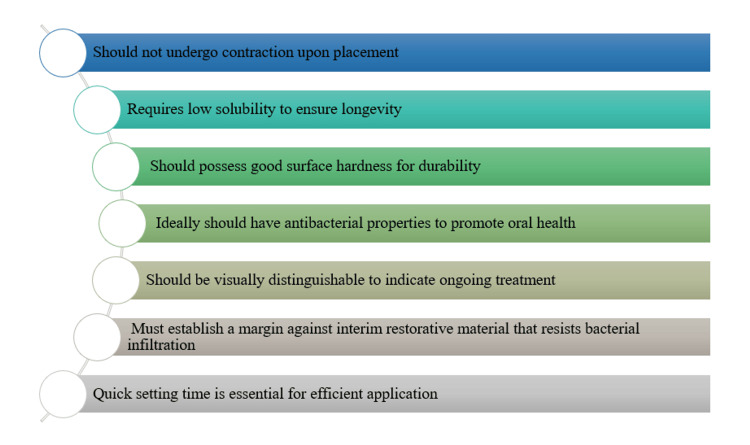
Ideal properties of temporary restorations

A restoration inserted into an endodontic access cavity is referred to by this term, often necessitated by cutting through an interim restoration. The choice of "temporary" is apt, as it implies a shorter duration than "interim", aligning with its provisional function until definitive treatment is completed. It differs significantly from the interim restoration in several aspects: it is utilized in reduced amounts, necessitating adequate strength in a minimal volume; its strength might be inferior compared to that of the interim material; it might exhibit a different colour and may not emphasize aesthetics in the same way as the interim material. Compared to the interim material, greater compressive strength is needed, but because of the different load distribution, less tensile strength is required. Despite lacking apparent retention or resistance form, the temporary restoration of the tooth must be adhered to; compatibility with interim restorative materials is essential; placing and removing it should be faster, easier, and cost-effective, making it generally cheaper than interim restorations [1]. Ideal properties of temporary restorations are as follows (Figure 2): should not undergo contraction upon placement; requires low solubility to ensure longevity; should possess good surface hardness for durability; ideally, should have antibacterial properties to promote oral health; should be visually distinguishable to indicate ongoing treatment; must establish a margin against interim restorative material that resists bacterial infiltration; quick setting time is essential for efficient application [1].

Interim restoration

**Figure 3:**
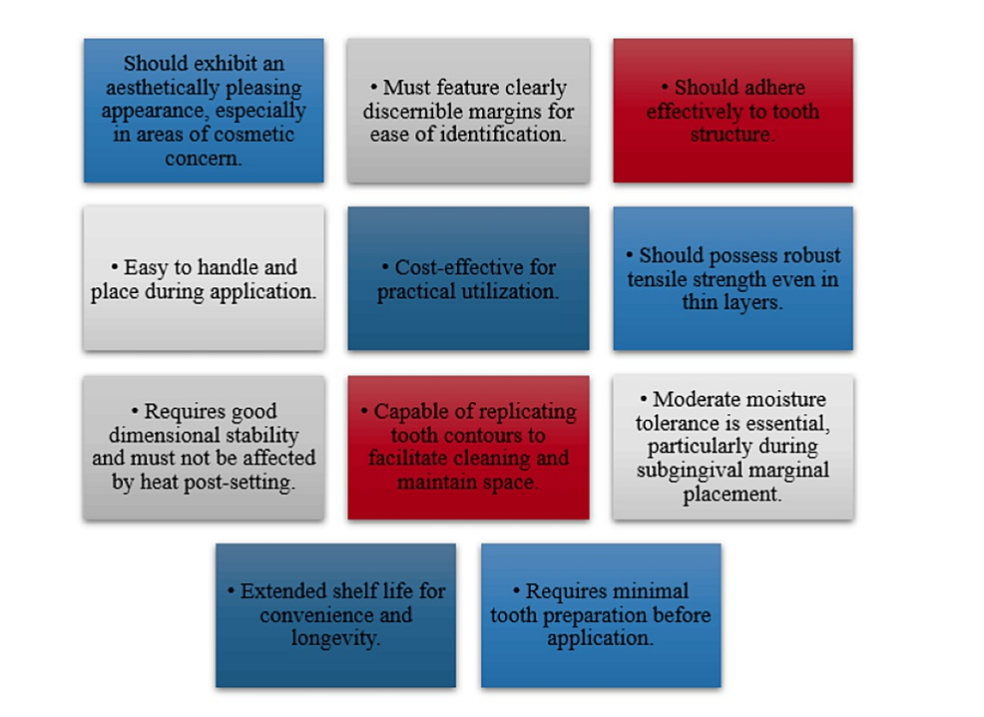
Ideal properties of interim restorations Reference: Anusavice [8].

This term describes a restoration placed in a tooth after removing previous restorations and all caries at the start of endodontic treatment. This restoration remains in position during the endodontic procedure and post-root canal filling until the final restoration is given [1]. The interim restoration is placed following the initial examination and assessment during the first endodontic appointment. Interim restorations used during endodontic therapy must not only avoid bacterial infiltration but also fulfil the following three requirements: enable the continued functionality of the tooth, enable the patient to uphold regular oral hygiene practices around the tooth, and ensure that the operator has sufficient access to canals. At present, there is no single material that satisfies all of these criteria. The ideal properties of interim restorations given by Anusavice in 1996 gave ideal interim restorations (Figure 3) [8].

Commonly used materials in endodontics are as follows: amalgam, glass ionomer cement (GIC), composite resins, resin-modified GIC, calcium sulphate-based filling material (e.g., Cavit, 3M ESPE, Seefeld, Germany), and reinforced interim restorative material in a zinc oxide eugenol (ZOE) base (e.g., IRM, Dentsply Caulk, Milford, CT) (Table 1).

**Table 1:** Properties of various dental cements that are used as temporary and interim restorative materials EBA: ethoxybenzoic acid; ZOE: zinc oxide eugenol.

Properties	Zinc phosphate cement	Polycarboxylate cement	Zinc oxide eugenol cement	EBA alumina bonded cement	Polymer-reinforced ZOE cement	Zinc oxide/calcium sulphate cement
Setting time	2.5 to 8 min	7-9 min	4-10 min	7-9 min	6-10 min	20-30 min
Compressive strength	104-119 Mpa	55 Mpa	45-55 Mpa	55-60 Mpa	49-71 Mpa	51 Mpa
Tensile strength	5.5 Mpa	6.2 Mpa	4.1 Mpa	4.1 Mpa	4.1 Mpa	8 Mpa
Stiffness/modulus of elasticity	13 Gpa	5 Gpa	2.5 Gpa	25 Gpa	25 Gpa	-
Solubility	0.06% wt	0.6% wt	0.4-1.5% wt	0.05% wt	0.04% wt	0.03% wt
pH	Initial pH: 2; 24 hours: 5.5	Initial: 3-4; 24 hours: 5-6	6.6-8	-	7	10.5-12

Presently, materials utilized embody a balance of various factors, yet clinical outcomes continue to show promise. A case report by Vail and Guba [9] highlights the significant interim restoration's role in preventing the ingress of microbes and consequently altering the environment to facilitate healing, even without complete endodontic treatment [9]. The study's authors described a patient who received emergency endodontic intervention but postponed receiving additional care for more than 2.5 years. Remarkably, despite minimal palliative measures, the interim restoration remained intact, and radiographic evidence revealed complete periapical healing. The diverse array of purposes, cavity design demands, and optimal properties for interim restorations often lead to the utilization of various materials. Despite this necessity, the dental literature frequently refers to "temporary materials" without a clear definition.

Gutta-Percha

This solidified juice is derived from the Isonandra gutta tree, an evergreen species in the Malay Peninsula and Archipelago. Chemically, gutta-percha comprises pure gutta, a hydrocarbon with the formula C10H16, albane (C40H64O3), and guttane. This composition bears a resemblance to rubber both in its origin and chemical structure. In practical applications, gutta-percha is rarely utilized in its pure form. Instead, additives like zinc oxide, magnesium oxide, white wax, calcium oxide, burgundy pitch, and carbon are often incorporated to enhance its desirable working characteristics. Its introduction to dentistry dates back to 1847 when Hill [10] pioneered its use, leading to its popularization under the moniker "Hill's stopping".

Gutta-percha-based materials undergo plasticization over a flame before being placed into the cavity. However, overheating can render the material crumbly [11]. One advantage of these materials is their easy placement, removal from the cavity, and good biological tolerance. Conversely, their low resistance to chewing pressure and the necessity for a dry cavity are notable disadvantages [12]. Initially, gutta-percha, temporary stopping, and base plate materials were among the first investigated, albeit showing less-than-optimal traits.

In experiments conducted by Parris et al. [13] using bacterial and dye penetration tests on extracted teeth, it was discovered that gutta-percha temporary fillings exhibited leakage under extreme temperatures ranging from 4°C to 60°C. In an in vivo investigation, access canals in teeth that had previously received effective root canal therapy were made again by Krakow et al. [14]. After 15 millilitres of irrigation with sodium hypochlorite and 15 millilitres of 0.067 M phosphate buffer with a pH of 7.2, the cavities were chemically disinfected. Cotton pellets were placed into the spaces under temporary fillings and remained there for at least one week. After this period, the pellets were removed and cultured anaerobically. Six out of eight samples temporized with gutta-percha demonstrated significant leakage.

Zinc Phosphate Cement

This cement was first introduced by Pierce in 1879 and has proven effective as a provisional restoration [15]. Zinc phosphate cement is one of dentistry's most commonly utilized materials, although its popularity is waning due to the emergence of newer modified cement.

Krakow et al. [14] conducted an in vivo study examining the efficacy of zinc phosphate cement in temporized access cavities. Their findings revealed that over two-thirds of cases showed no evidence of leakage. Bobotis et al. [16] assessed microleakage using the fluid filtration method. According to their research, there were situations where the microleakage in zinc phosphate cement was similar to that of an unbroken crown. However, there was evident leakage in some samples temporized with zinc phosphate cement.

Polycarboxylate Cement

Smith in 1968 introduced polycarboxylate cement. This cement exhibits strength and manipulative characteristics akin to phosphate cement while showcasing a low potential for irritation when utilized correctly [17]. Their key benefit lies in their adhesive quality, making them advantageous for various dental procedures.

In vitro studies assessing the effectiveness of this material as a temporary restoration have produced inconsistent results. Marosky et al. [18] discovered that polycarboxylate cement provided the least favourable seal compared to other materials such as Temp Seal (GDT, Beer Sheva, Israel), Cavit, ZOE, zinc phosphate cement, and intermediate restorative material (IRM). Even after thermocycling, polycarboxylate cement at a powder/liquid (P:L) ratio of 2:14 was not substantially different from Cavit-G, according to Pashley et al. (1988) [19], utilizing a fluid filtration approach. Despite these studies, this cement is not commonly used or advised due to the lack of well-established clinical effectiveness for endodontic temporization.

Calcium Sulphate/Zinc Oxide Preparations

Cavit is a pre-mixed temporary filling material and contains colours, polyvinyl chloride acetate, glycol acetate, calcium sulphate, resins of polyvinyl acetate, and zinc oxide [20]. Triethanolamine is also included. Cavit absorbs water and exhibits a high coefficient of linear expansion because it is hygroscopic. Its linear expansion nearly doubles that of ZOE, contributing to its exceptional sealing ability [20]. The coefficient of linear expansion for Cavit is 14.2% per degree Celsius (°C-1), while for ZOE, it is 8% per degree Celsius (°C-1). Other commercial products in this category include Cavit™ (ESPE, Seefeld, Germany), Caviton (GC Dental, Tokyo, Japan), and Coltosol (Coltene, Cuyahoga Falls, OH).

Cavit is produced in three variations: Cavit™, which is pink; Cavit™ W, which is white; and Cavit™ G, which is grey, each manufactured for distinct clinical applications. Cavit™ has high surface hardness with the hardest set, which is used to fill occlusion-loaded restorations after endodontic treatments temporarily. Cavit™ W has reduced final hardness and increases adhesion, which provides increased adhesion for application after endodontic therapies. Cavit™ G has the softest set and is ideal for the temporary setting of inlay preparations because it may be obliterated without burs. Cavit™ and Cavit™ W are materials composed of zinc oxide and zinc sulphate in distinct concentrations, accompanied by different additives. As stated by the manufacturer, these variances lead to disparate final hardness levels for Cavit™ and an enhanced adhesion capacity for Cavit™ W [21].

The sealing effectiveness of Cavit™ has been extensively studied across a range of research settings, including laboratory and real-world scenarios, typically resulting in positive outcomes. A study by Webber et al. [22] explored the minimum Cavit™ thickness to inhibit methylene blue dye leakage in vitro. Their findings revealed that a minimum of 3.5 mm of the material was essential to prevent such leakage. Furthermore, in a research conducted by Barkhordar and Stark [23] where they compared the sealing capabilities of Cavit™ in divergent class I or parallel cavity preparations, it demonstrated superior effectiveness over IRM and temporary endodontic restorative material (TERM), respectively. Nevertheless, neither the influence of the two cavity designs nor the difference between Cavit™ and TERM reached statistical significance.

In a 1977 study, Krakow [14] used Cavit™ to temporize anterior access openings. The results showed that 27 out of 32 teeth had no or minimal microleakage. Microleakage only occurs in 15% of cases. Another study by Beach et al. in 1996 [24] revealed that having a Cavit™ thickness of 4 mm offered the most effective seal over a three-week temporization period, outperforming IRM and TERM. In a study by Jacquot in 1996 [25], it was observed that the dimensional stability and hardness of Cavit™ G, Cavit™ W, and Cavit™ decreased. Specifically, Cavit™ and Cavit™ W exhibited similar levels of water tightness, which were notably superior to those of Cavit™ G. These variations in sealing efficacy were attributed to the differences in resin content and, subsequently, the hardness and setting characteristics between Cavit™ G and Cavit™ W.

Cavidentin, produced by Laszlo Laboratories in Netanya, Israel, is another calcium sulphate-based material closely resembling Cavit™. However, it varies due to the inclusion of antiseptic, which is thymol and potassium aluminium sulphate as a catalyst. A study conducted by Tamse et al. in 1982 [26] discovered that Cavidentin, with a thickness of 5 mm, exhibited superior sealing abilities compared to Cavit, Kalzinol (Dentsply, York, PA), and IRM. Cavidentin exhibited nearly equal effectiveness to Cavit™ G in providing a reliable seal.

The ingredients of Coltosol, produced in Mahwah, NJ by Coltene Whaledent, are calcium sulphate hemihydrate, zinc oxide, and zinc sulphate. Upon contact with moisture, the surface of Coltosol undergoes hardening within 20-30 minutes, allowing for mastication pressure to be applied after two to three hours, as the manufacturer recommends. Primarily intended for short-term temporization lasting no more than two weeks, Coltosol has not been explicitly evaluated for use as a temporary restoration in endodontics [27].

From a clinical standpoint, Cavit and its variants offer several advantages. They are easy to handle, readily available in pre-mixed paste form, and can be removed from access cavities with ease once set. Moreover, it is evident that Cavit™ effectively seals access cavities between appointments. However, it is important to note some significant drawbacks: slow-setting reaction, wear resistance, hardness, and tendency to deteriorate over time [20,28]. Due to these factors, Cavit™ is frequently recommended for temporary filling in cavities for the short term. To overcome its shortcomings, experts recommend adopting a dual-seal strategy, incorporating Cavit™ as the inner layer and IRM as the outer layer. This approach is suggested to offset the less favourable physical attributes of Cavit™. This dual-seal technique has demonstrated superior adhesion to dentine compared to using IRM only [29].

Preparations Containing Zinc Oxide and Eugenol

Numerous temporary restorations rely on ZOE formulations, with or without reinforcement. The conventional use of plain ZOE, with a P:L ratio of 4:1, often yields a subpar initial seal, albeit showing some enhancement after a week [19]. Alternatively, adopting a low P:L ratio (2:1) offers improved initial sealability, although this seal might slightly deteriorate over time [19].

In their study, Marosky et al. (1977) [18] discovered that simple ZOE temporary cement exhibited lower efficacy in inhibiting leakage of radioactive tracer in comparison to Temp Seal and Cavit™. However, it demonstrated superiority over polycarboxylate cement IRM and zinc phosphate cement [18]. Commercial products based on ZOE, such as Dentemp (Majestic Drug Co., Bronx, NY) and Kalsogen Plus (De Trey, Dentsply, York, PA), have undergone testing and comparison with substitute materials. Noguera and McDonald (1990) [30] observed that it was almost as successful as IRM. Similarly, Mayer and Eickholz (1997) [31] discovered that Kalsogen was not as successful as Cavit™ and TERM in inhibiting dye penetration following mechanical loading and thermocycling.

Kalzinol is a ZOE-based cement with its compressive strength almost doubled by adding 2% by-weight polystyrene polymer reinforcement. Studies using an electrochemical method to measure microleakage have demonstrated that this cement provides better sealing qualities than Cavit™ W and is almost as good as GIC used in unconditioned cavities [32]. IRM is a ZOE cement incorporating polymethyl methacrylate for reinforcement. The restoration's hardness, abrasion resistance, and compressive strength are all improved by this strengthening [33-34]. Per the manufacturers' instructions, IRM is advised for temporary cavity restorations for up to one year, with a P:L ratio of 6:1. Adhering to these instructions often yields a seal that might be suboptimal but generally enhances the physical properties. Reducing the amount of powder used yields a superior seal. Moreover, a soft mixture displays heightened antibacterial activity through hydrolysis, leading to increased eugenol release that could potentially hinder bacterial colonization in the event of leakage [35].

Numerous in vivo and in vitro research have compared and assessed IRM with various temporary restorative materials, with inconsistent outcomes. In an in vivo study conducted by Beach et al. (1996) [24], IRM demonstrated similar efficacy to Cavit™ in temporizing class I access cavities in human teeth over three weeks when applied with a 4-millimeter thickness. Conversely, Blaney et al. (1981), in their in vitro study, found that IRM, when allowed to set adjacent to calcium hydroxide-containing CMCP (calcium hydroxide, methylcellulose, pectin), exhibited significantly better resistance to *Proteus vulgaris* penetration compared to Cavit™ set under similar conditions. Notably, these findings are intriguing given that the surface hardness of IRM is reduced by CMCP while having no impact on Cavit™ hardness. Most in vivo and in vitro studies utilizing bacterial assessments have consistently shown nearly equivalent or superior sealing performance with IRM or ZOE compared to Cavit™, as documented by Parris et al. in 1964, Krakow et al. in 1977, Blaney et al. in 1981, Beach et al. in 1996, and Barthel et al. in 1999 [13,14,24,33,36]. Drawing from the preceding discourse and the findings of in vivo investigations, it is evident that ZOE temporary restorative materials, such as IRM, possess the capacity to offer sufficient resistance against microleakage throughout endodontic treatment, mainly when a reduced P:L ratio is used.

Glass Ionomer Cement

GICs have been extensively explored for their applications in endodontics. Several studies have examined their efficacy as temporary restorations during endodontic therapy, consistently yielding positive outcomes. Their adhesion mechanisms elucidate the acceptable sealing ability of GIC. An in vitro investigation conducted by Lim in 1990 [32], utilizing an electrochemical technique, unveiled that GIC, when inserted into unconditioned cavities, exhibited nearly equivalent effectiveness compared to Kalzinol and outperformed Cavit™ W following a one-month experimental duration [32]. When compared to Cavit™, GIC and Cavit™ poured over a Cavit foundation over one month, Barthel et al.'s (1999) study showed that GIC applied either alone or over an IRM basis demonstrated a noticeably enhanced seal against *Streptococcus mutans* penetration [36].

The antibacterial efficacy of these materials stems from fluoride release, low pH, and the presence of specific cations like strontium and zinc in certain formulations. As a result, GICs are considered appropriate for use as temporary restorative materials in endodontic procedures and can even be utilized in situations requiring prolonged temporization. However, challenges arise due to cost, setting time, and difficulty distinguishing glass ionomers from surrounding tooth structures during removal.

Composite

For endodontic operations, TERM is a recently developed temporary restorative option. TERM comprises urethane methacrylate polymers, inorganic radiopaque fillers, organic pre-polymerized fillers, colours, and initiators. It is a single-component light-curable resin. TERM has polymerization shrinkage like other composite resins, around 2.5% of its volume.

Utilizing the fluid filtration approach and thermocycling, researchers examined the minimal thickness needed for successful cavity sealing with TERM in an in vitro study by Hansen & Montgomery in 1993. After five weeks and thermocycling, their results showed that TERM thickness between 1 and 3 millimetres was just as good at providing seal as a thickness of four millimetres [37].

The material has been scrutinized in various in vivo and in vitro investigations, yielding conflicting results. Teplitsky and Meimaris (1988) discovered in their in vitro study that TERM provided a satisfactory, marginal seal in 33.3% of cases, compared to 91.7% for Cavit™. Additionally, thermocycling did not adversely affect the sealability of Cavit™ but increased microleakage incidence with TERM [38]. Conversely, Melton et al. (1990) [39] found that when used to seal etched and non-etched cavities without thermocycling, TERM demonstrated 67% sealing ability compared to 100% for Cavit™. According to Beach et al. (1996), when applied with a 4-millimeter thickness for a three-week temporization period, TERM shows less efficiency in class I cavities than Cavit™ and IRM. Nonetheless, TERM and Cavit™ have demonstrated comparable efficacy in multiple tests. TERM was discovered to have a marginal seal superior to Cavit™. In contrast, IRM's physical qualities were thought to be superior to those of TERM and Cavit™, according to the authors Barkhordar and Stark [23].

## Conclusions

A comprehensive examination of the studies cited is essential as they provide valuable insights into clinical scenarios. Persistent periapical disease following endodontic treatment mainly arises from bacterial presence within the root canal system. Therefore, during and after treatment, careful attention is needed to ensure that all bacteria are removed and to prevent any more invasion of the tooth. As such, it is imperative that the significance of interim and provisional restorations be appropriately acknowledged, highlighting their central place in endodontic treatment regimens.
